# A circadian rhythm-related lncRNA signature correlates with prognosis and tumor immune microenvironment in head and neck squamous cell carcinoma

**DOI:** 10.1007/s12672-024-01181-z

**Published:** 2024-07-25

**Authors:** Hongyu Zheng, Qiuyue Li, Kai Yang

**Affiliations:** 1 Department of Oral and Maxillofacial Surgery, The First Affiliated Hospital of Chongqing Medical University, No.1, Youyi Road, Yuzhong District, Chongqing, 400016 China; 2 Department of Emergency Medicine, The Second Hospital of Tianjin Medical University, No.23, Pingjiang Road, Hexi District, Tianjin, 300211 China

**Keywords:** Circadian rhythm, lncRNA, Squamous cell carcinoma, Prognostic signature, Immune microenvironment

## Abstract

**Objective:**

To investigate circadian rhythm-associated long non-coding RNA (lncRNA) signatures in predicting prognosis, metabolism, and immune infiltration in Head and Neck Squamous Cell Carcinoma (HNSC).

**Methods:**

HNSC samples were collected from the TCGA database. A signature was constructed using Cox regression and Least Absolute Shrinkage and Selection Operator (LASSO) methods. The immune cell infiltration was analyzed using CIBERSORT, ssGSEA, and MCPcounter. The RT-qPCR was used to detect the expression of signature lncRNAs.

**Results:**

A signature comprising 8 lncRNAs was constructed. The constructed signature demonstrated good prognostic prediction capability for HNSC. A nomogram encompassing risk score accurately predicted the long-term OS probability of HNSC. The infiltration levels of T cell, B cell and Macrophages were significantly higher in the high-risk group than in the low-risk group. Cluster analysis showed that the signature lncRNAs could classify the HNSC samples into two clusters. The RT-qPCR suggested that the expression of lncRNAs in signature was consistent with the data in TCGA.

**Conclusion:**

The circadian rhythm-associated lncRNA signature has potential as a prognostic indicator for HNSC. It exhibits associations with metabolism, immune microenvironment, and drug sensitivity, thereby providing valuable insights for informing the treatment of HNSC.

**Supplementary Information:**

The online version contains supplementary material available at 10.1007/s12672-024-01181-z.

## Introduction

Head and neck tumors are among the most prevalent types of cancer worldwide, and HNSC represents the predominant form of head and neck cancer. Annually, approximately 600,000 new cases of HNSC are reported globally [1, 2]. The prognosis for patients with locally advanced HNSC is generally poor, with a high recurrence rate of 40–50% even after multimodal therapy [3]. Therefore, it is crucial to identify reliable prognostic biomarkers for HNSC. With advancements in molecular biology, investigating prognostic markers at the molecular level holds promise for achieving more accurate individualized survival predictions. In recent years, there has been growing interest in exploring the predictive role of molecular prognostic markers in HNSC prognosis [4–6]. Additionally, numerous bioinformatics analyses related to HNSC have been conducted utilizing the TCGA and GEO databases [7, 8].

Almost all organisms depend on the biological clock to maintain the circadian cycle of physiological processes such as sleep, diet, mood, metabolism and immunity [9]. In recent years, a growing number of studies have explored the relationship between biological rhythms and cancer. Disrupted expression of circadian rhythm-related genes has been detected in various tumor types [10]. Similarly, altered expression of biological clock genes has been observed in studies focusing on HNSC, impacting tumor progression and apoptosis [11, 12]. Circadian rhythm is an indispensable biological mechanism in tumorigenesis and progression, potentially contributing to prognosis prediction and treatment strategies for HNSC.

lncRNA refers to a class of non-coding RNAs comprising at least 200 nucleotides. lncRNA is now thought to play a key role in many tumor processes and in the development of head and neck squamous cell carcinoma [13]. These lncRNAs exert influence on tumor progression by modulating the epithelial-mesenchymal transition process in HNSC cells [14]. Additionally, they can serve as diagnostic markers and therapeutic targets in HNSC [15]. Moreover, lncRNAs have been identified as immunomodulators in tumors [16], displaying associations with the tumor microenvironment in HNSC and impacting the efficacy of immunotherapy [17].

Circadian rhythm-associated lncRNAs, their diagnostic potential, and therapeutic targeting in HNSC remain relatively unexplored areas of research. Therefore, in-depth investigation into these circadian rhythm-associated lncRNAs can enhance our understanding of their significance in HNSC diagnosis and immunotherapy. This, in turn, may present new avenues for precise treatment strategies and individualized management approaches.

## Materials and methods

### Data acquisition and screening

The RNA transcriptome datasets, including counts and FPKM format data, along with related clinical information, were obtained from the UCSC-XENA database (https://xenabrowser.net/datapages/). For the differential expression analysis, the counts format data were utilized, whereas the FPKM data were employed for signature construction and prognostic analysis. To minimize statistical bias, rigorous screening of samples and genes was performed, adhering to the following criteria: (1) only samples derived from primary tumors and normal tissues were included in the analysis; (2) samples lacking information on survival and overall survival (OS) of less than 30 days were excluded; and (3) RNA transcripts exhibiting expression values of 0 in more than half of the samples were removed, ensuring the reliability and accuracy of downstream analyses.

### Acquisition of circadian rhythm genes and screening of related lncRNAs

A comprehensive collection of 31 circadian rhythm-related genes was obtained from the KEGG database’s Circadian rhythm pathway (https://www.kegg.jp/) and supplemented by relevant study [18] (Supplementary Table 1). Subsequently, the lncRNA expression data were extracted from the RNA expression data, and differentially expressed lncRNAs (DElncRNAs) were identified using the DESeq2 package in R language, following the criteria for differential expression (|Log2foldchange|> 1, adjust *P* value < 0.05). To analyze the correlation between DElncRNAs and the expression of the 31 circadian rhythm-related genes, a Pearson correlation analysis was performed (correlation coefficient > 0.4, *P* < 0.01).

### Construction and validation of prognostic risk assessment signature

The TCGA primary tumor samples were randomly divided into training and validation sets in a 1:1 ratio. In the training set, lncRNAs associated with OS were screened from the relevant lncRNAs using univariate COX regression analysis. To preclude the risk of overfitting, a secondary screening of prognostic-related lncRNAs was performed using the least absolute shrinkage and selection operator (LASSO) regression analysis. This iterative process yielded a subset of lncRNAs and their respective LASSO regression coefficients, which were subsequently utilized in constructing the risk scoring signature. The risk scoring signature equation was as follows:

Risk score = $$\sum_{i=1}^{n}(Coefi*Xi)$$

The risk score of each sample was calculated according to the formula, and the samples were divided into high-risk and low-risk groups according to the median risk score, and the difference in OS between high- and low-risk groups was analyzed using the KM method. ROC curve analysis was performed using the ROCR and timeROC packages as a way to assess the accuracy of the prediction signature.

To verify the accuracy of the risk scores, the risk scores of each sample were calculated according to the formula in the validation set and the complete data set, and the samples were divided into high-risk and low-risk groups according to the bit risk scores in the validation set and the complete data set, and the differences in OS were analyzed using the KM method. ROC curve analysis was performed to assess the accuracy of the prediction signature.

### Correlation of risk signature score with prognostic and clinicopathological parameters

Univariate COX regression analysis was conducted to examine the correlation between the risk score, gender, age, T-stage, N-stage, tumor grade, tumor stage, and OS. For indicators with a significance level of *P* < 0.05, multivariate COX regression analysis was performed to identify independent risk factors influencing prognosis. The tumor samples were subgrouped based on gender, age, T-stage, N-stage, tumor grade, and tumor stage. Then, KM analysis was used to analyze whether there were differences in OS between high-risk and low-risk patients in each pathological parameter subgroup.

### Construction of prognostic nomogram

To improve the accuracy of prognosis prediction for patients with HNSC, we used stepwise regression methods to identify Prognosis related clinical indicators. Subsequently, a nomogram model was constructed using these selected indicators. The ability of the nomogram model to predict prognosis was assessed using time-dependent ROC analysis. Also, the predictive effect of the predictive nomogram at 1, 3 and 5 years was assessed using calibration curves. Decision Curve Analysis (DCA) was used to assess the predictive effect of the nomogram plot and the parameters that make up the nomogram on prognosis.

### GO, KEGG and GSVA analysis

Samples were divided into high-risk and low-risk groups according to risk scores, and the DEseq2 method was used to analyze differential gene expression between high- and low-risk groups (|Log2foldchange|> 1, adjust *P* value < 0.05). To further explore the biological functions and pathways associated with these differentially expressed genes, we conducted gene ontology (GO) analysis and Kyoto Encyclopedia of Genes and Genomes (KEGG) analysis using the clusterProfiler package [18]. *P* < 0.05 was recognized as a significant pathway.

The GSVA genomic enrichment method was used to assess the changes in pathway enrichment levels in HNSC samples [19]. The gene sets “h.all.v7.5.1.symbols.gmt” and “c2.cp.kegg.v7.5.1.symbols.gmt” downloaded from Molecular Signatures Database (MSigDB; https://www.gsea-msigdb.org/) were used as references. The R package “GSVA” was used to calculate the enrichment fraction of different biological pathways in HNSC samples. The “limma” package was then used to analyze the differences in pathways in the high-risk and low-risk groups. *P* < 0.05 was recognized as a significant pathway.

### Analysis of differences in metabolic pathway enrichment levels

The gene sets of 36 metabolism-related pathways were obtained by MSigDB and KEGG as well as in a previous study [20]. The enrichment level of each pathway was analyzed using the ssGSEA method, and the difference in the enrichment level of pathways in high and low risk groups was analyzed using the wilcox method.

### Immune cell infiltration and immune microenvironment analysis

The immune infiltration status of each tumor sample was calculated based on the expression data of each TCGA sample, including CIBERSORT, ssGSEA and MCPcounter methods, and the differences in immune cell level infiltration between high and low risk groups were analyzed using the wilcox method. Then, the relationship between each lncRNA expression level in the signature and the risk score with each immune cell infiltration level was analyzed using the 'psych' package. Immune microenvironment metrics were assessed for each sample using the EATIMATE method, including StromalScore, ImmuneScore, ESTIMATEscore, and TumorPurity, and the differences in these metrics between the high- and low-risk groups were analyzed using the wilcox method.

### The impact of risk scores on immunotherapy

Seventy-five genes for immune-related markers were obtained from a previous study [21], and the difference in expression of immune-related markers between high- and low-risk groups was analyzed using the wilcox method.

According to Xu et al. [22], anti-tumor immunity was divided into anti-cancer immune states of a seven-step cancer immune cycle. The enrichment scores of each immune event in each sample were analyzed using the ssGSEA method, and the correlation between the risk score and each immune event was analyzed using Pearson correlation analysis. In addition, wilcox was used to analyze the differential expression of immune checkpoint inhibitors in high and low risk groups, including PDCD1, CD274, CTLA4, HAVCR2. LAG3, TIGIT, and IDO1.

### Tumor mutation burden(TMB) and Drug sensitivity analysis

Tumor mutation data (TCGA.HNSC.varscan.DR-10.0.somatic) were obtained from the TCGA database. The tumor mutational load (TMB) was calculated for each sample based on the somatic mutation data. The samples were then divided into high and low mutation load groups based on the median mutation load values, and the samples were divided into four types by combining the high and low risk groups to analyze the survival differences in the four conditions. Differences in OS of the four groups were analyzed using the KM method.

The half-maximal inhibitory concentration (IC50) values of the drugs cisplatin, docetaxel, paclitaxel, and 5-Fu, the main drugs used clinically for the treatment of HNSC, were analyzed in each sample using the ‘oncoPredict’ R package, and the difference in IC50 between the high-risk and low-risk groups was analyzed using the Wilcox method. Further Pearson correlation analysis was used to analyze the correlation between risk scores and signature lncRNA and drug IC50.

### Clustering analysis based on signature lncRNAs

The ConsensusClusterPlus method was used to cluster the primary tumor samples using the expression values of the lncRNAs in the risk score signature [23], and the effect of cluster analysis on the samples after cluster analysis was analyzed using the principal component analysis (PCA) method. The Sankey diagram method was used to analyze the sample consistency between the two subclasses of cluster analysis and the two groups of high and low risk.

### Cell culture and quantitative real-time PCR

Normal human oral keratinocytes (HOK) were purchased from Shanghai Huiying Biotechnology Co., Ltd., CAL27, SCC25 and SCC15 were purchased from Shanghai Tongpai Biotechnology Co., Ltd. All cell lines were cultured with DMEM containing 10% fetal bovine serum (FBS, S711-001S, Uruguay) and 1% penicillin–streptomycin in a 37 °C, 5% CO_2_-based incubator.

RNA was extracted with RNAiso Plus (9180, TaKaRa, Japan). PrimeScript RT reagent Kit with gDNAEraser (Perfect Real Time) (RR047A, TaKaRa) was used to reverse transcribe RNA into cDNA. The RT-qPCR was performed according to the instructions of TB Green Premix Ex TaqTM II (RR820A, TaKaRa). The primer sequences are shown in Supplementary Table 2. The mRNA expression of each gene was calculated using the 2^−∆∆Ct^ method.

## Results

### Screening of DElncRNAs and circadian rhythm-related lncRNAs

**Fig.1:**
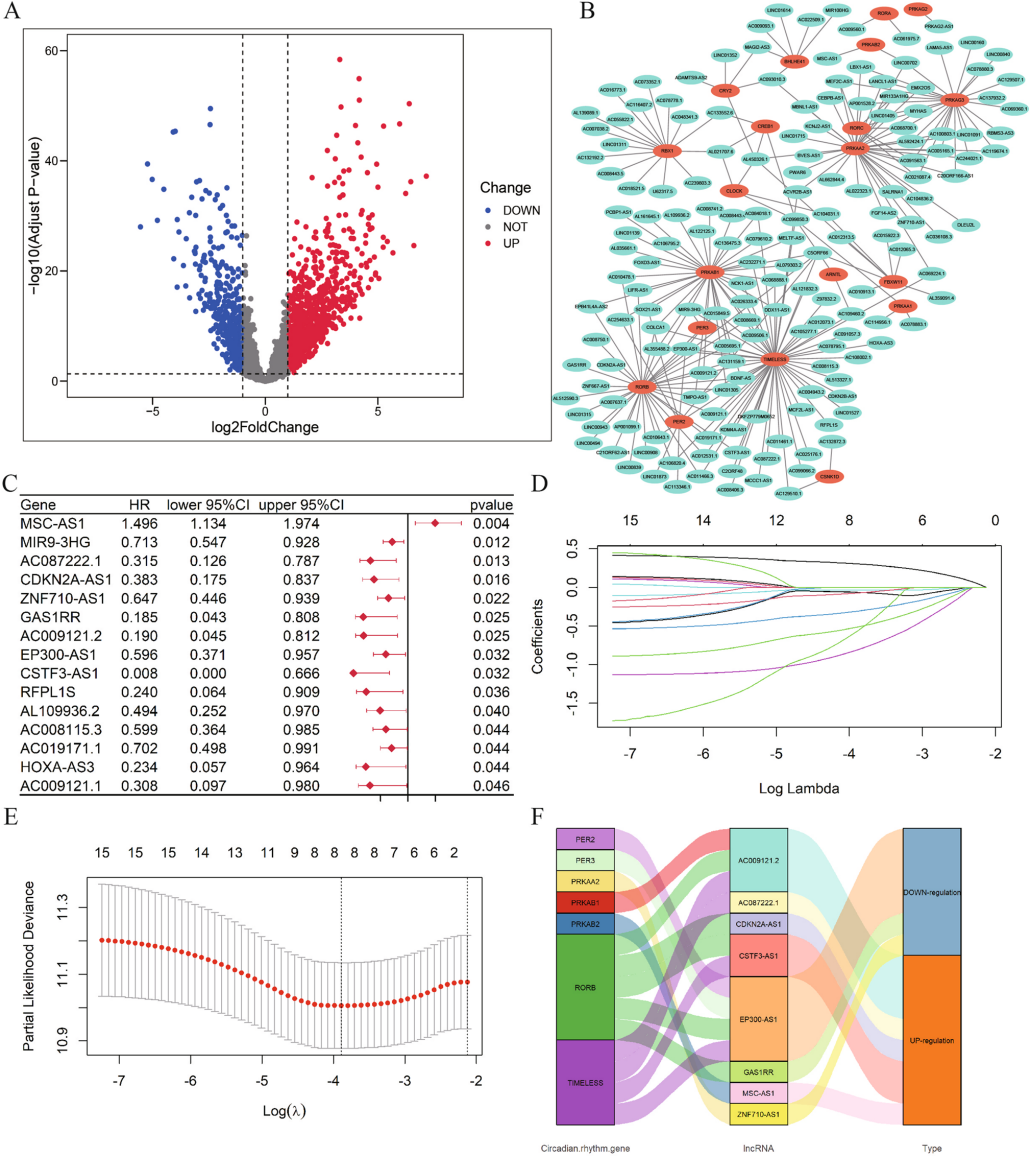
Circadian rhythm related lncRNA screening and signature lncRNA screening. **A**. Volcano plot of differential expression of lncRNAs in TCGA; **B**. Plot of lncRNA correlation with circadian rhythm genes; **C**. Univariate COX regression analysis of DElncRNAs associated with circadian rhythms; **D**. LASSO coefficient profiles; **E**. LASSO analysis of lncRNA screening; **F**. Sankey diagram of lncRNA correlation with circadian rhythm genes and their high and low expression in the signature

After sample screening, 486 primary tumor tissues and 44 normal tissue samples from TCGA data were included in the study (Supplementary Table 3). A total of 1457 lncRNAs were differentially expressed, of which 1040 lncRNAs were highly expressed in tumor tissues and 417 lncRNAs were lowly expressed (Fig. 1A). Based on the expression levels of DElncRNAs and 31 circadian genes, a total of 169 DElncRNAs were correlated with the expression of circadian genes after Pearson correlation analysis screening (*P* < 0.05) (Fig. 1B).

### Prognostic risk signature development and validation

The 486 tumor samples were randomly divided into a training set (245 cases) and a validation set (241 cases). There were no significant differences observed in the clinicopathological parameters between these two datasets (Supplementary Table 4). In the training set, 15 lncRNAs associated with circadian rhythm were associated with OS in HNSC (*P* < 0.05) (Fig. 1C). After LASSO regression, eight lncRNAs associated with circadian rhythms in HNSC were screened (Fig. 1D-E), of which five were highly expressed and three were lowly expressed (Fig. 1F).The formula used for calculating the risk score was as follows:

Risk Score = 0.3043 × MSC-AS1-0.5141 × AC087222.1-0.3078 × CDKN2A-AS1-0.0372 × ZNF710-AS1-0.8091 × GAS1RR-0.0636 × AC009121.2–0.0730 × EP300-AS1- 0.5655 × CSTF3-AS1.

**Fig.2:**
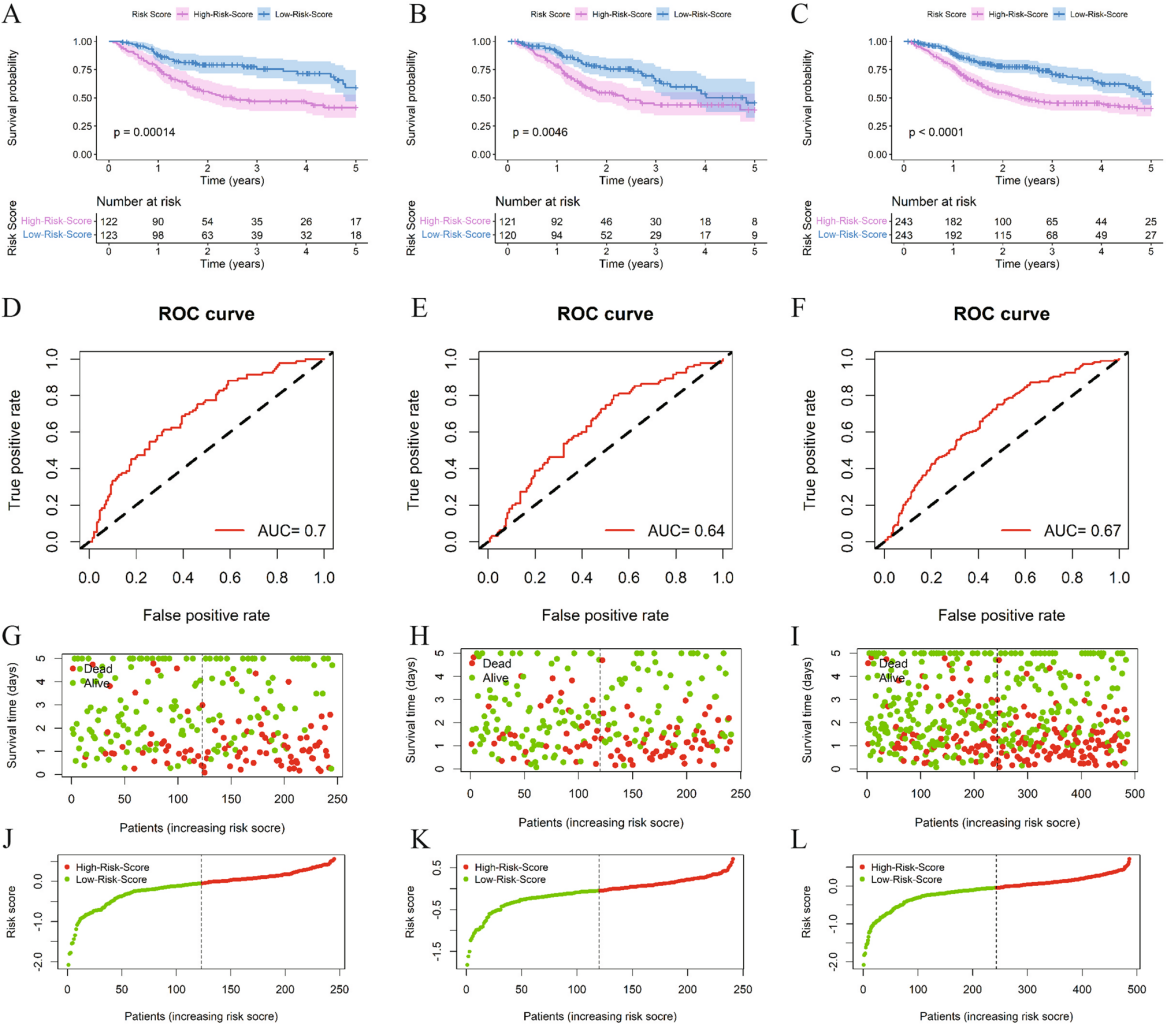
Predictive effect of the signature on prognosis in the training set, the test set and the complete set. **A**–**C**. KM curves for OS survival analysis between high and low risk score groups in the training set, validation set, and complete set; **D**–**F**. ROC curves for predicting prognosis by risk scores in the training set, validation set, and complete set; **G**–**I**. Survival status plots in the training set, validation set, and complete set; **J**–**L**. Distribution of risk scores in the training set, validation set, and complete set

Patients in the high-risk group had significantly shorter OS compared with the low-risk group (*P* < 0.05) (Fig. 2A). This trend was also observed in both the validation set and the complete dataset (*P* < 0.05) (Fig. 2B,C). This suggests that the high-risk score is a poor prognostic factor. The ROC curve analysis showed that the area under the curve (AUC) was 0.70 (sensitivity 0.61, specificity 0.68), 0.64 (sensitivity 0.80, specificity 0.47), and 0.67 (sensitivity 0.75, specificity 0.52) for the training set, validation set, and complete data set, respectively (Fig. 2D–F). The distribution plot of the signature score showed that as the risk score increased in the training set, validation set, and complete dataset, the proportion of patients in the high-risk group also increased, as did the level of mortality, while survival time decreased (Fig. 2G–L). It showed that the risk score signature had an accurate predictive effect on OS in HNSC patients.

In addition, the results of KM analysis in clinicopathological parameter subgroups suggested that OS was significantly shorter in the age ≤ 60 years group, age > 60 years group, male group, female group, T1-T2 group, T3-T4 group, N1-N3 group, G1-G2 group, G3-G4 group, Tumor stage I-II and Tumor stage III-IV group in all high-risk patients (*P* < 0.05). (Supplementary Fig. 1).

Among signature lncRNAs, the OS was significantly shorter in patients with high expression of MSC-AS1 and in patients with low expression of AC087222.1, CDKN2A-AS, ZNF710-AS1, GAS1RR, AC009121.2, EP300-AS1 and CSTF3-AS1 (*P* < 0.05) (Supplementary Fig. 2 A, B), which is consistent with this result of the coefficient of lncRNA in the signature.

### Risk score is an independent risk factor for HNSC prognosis

**Fig.3:**
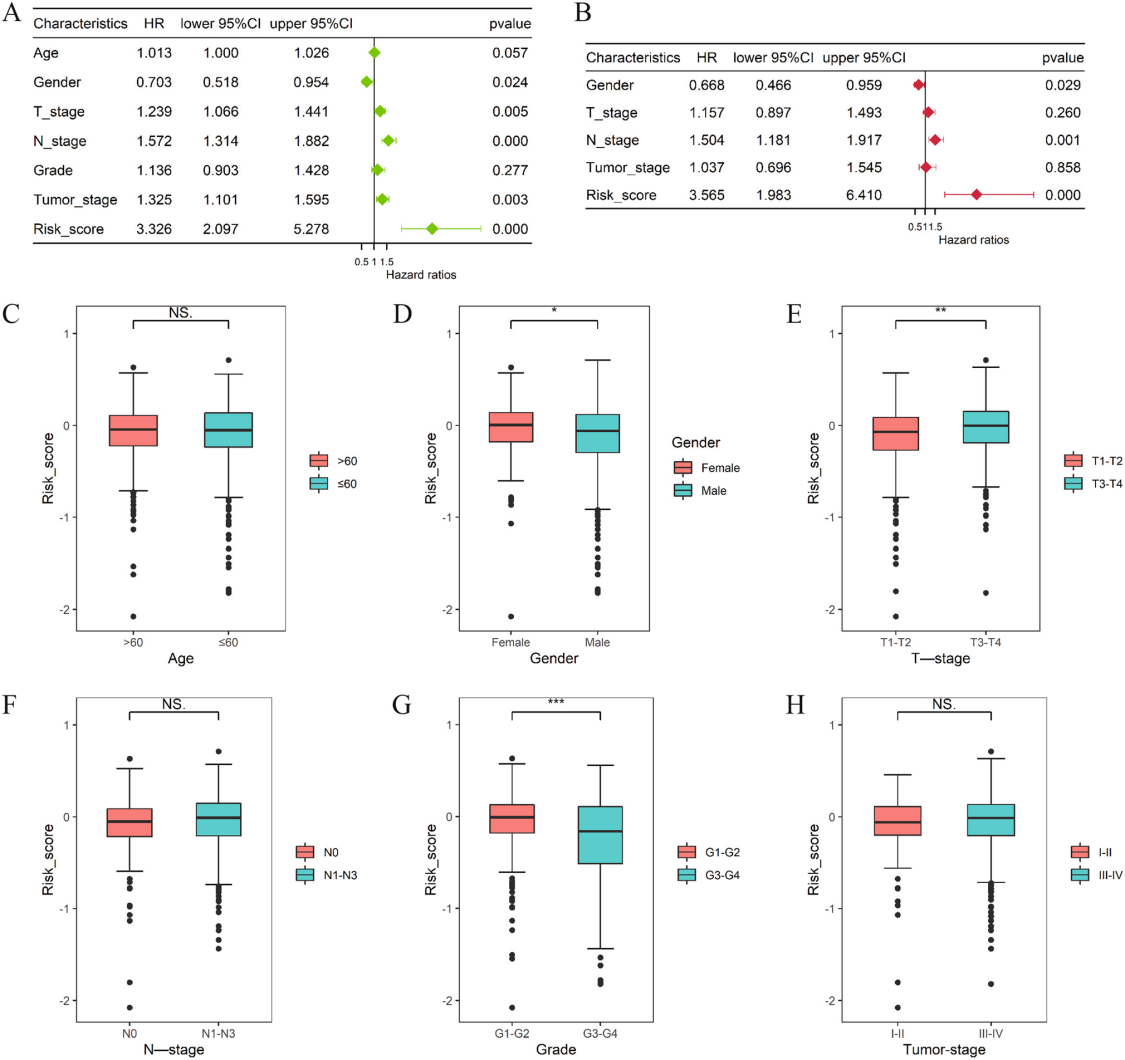
Risk score is an independent risk factor for HNSC prognosis. **A**. Forest plot of univariate COX regression analysis; **B**. Forest plot of multivariate COX regression analysis; **C**. Difference in risk scores between different age groups; **D**. Difference in risk scores in different gender groups; **E**. Difference in risk scores in different T-stage groups; **F**. Difference in risk scores in different N-stage groups; **G**. Difference in risk scores in different Grade groups; **H**. Difference in risk scores in different Tumor stage groups

Univariate COX analysis showed that gender, T-stage, N-stage, tumor pathological grade and risk score were risk factors for HNSC prognosis (*P* < 0.05) (Fig. 3A). Multivariate COX analysis revealed that gender, N-stage, and the risk score were independent risk factors for HNSC prognosis (*P* < 0.05) (Fig. 3B) (Table 1). Risk scores were higher in the female group than in the male group, in the T3-T4 group than in the T1-T2 group, and in the G1-G2 group than in the G3-G4 group (Fig. 3C–H).

**Table 1:** Univariate and multivariate COX regression analysis

Characteristics	Univariate cox				Multivariate cox			
	HR	pvalue	CI-lower	CI-upper	HR	pvalue	CI-lower	CI-upper
Age	1.0129	0.0566	0.9996	1.0263	–	–	–	–
Gender	0.703	0.0238	0.518	0.9542	0.6682	0.0286	0.4657	0.9588
T stage	1.2392	0.0053	1.0658	1.4407	1.1574	0.2601	0.8974	1.4927
N stage	1.5722	< 0.001	1.3137	1.8815	1.5043	0.001	1.1805	1.9169
Grade	1.1357	0.2769	0.9029	1.4284	–	–	–	–
Tumor stage	1.3251	0.0030	1.1006	1.5955	1.0371	0.8578	0.696	1.5454
Risk score	3.3264	< 0.001	2.0966	5.2777	3.5648	< 0.001	1.9826	6.4098

*HR* Hazard.Ratio, *CI* confidence interval

### Nomogram construction and calibration curve

**Fig.4:**
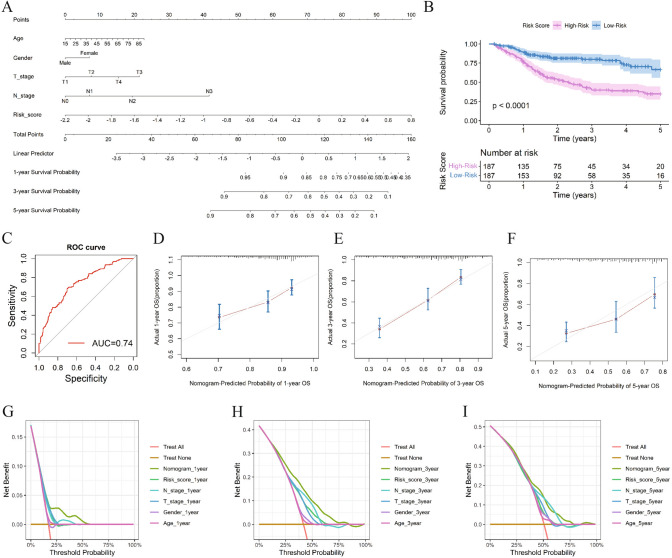
Nomogram modeling and validation. **A**. Nomogram plots; **B**. KM curves for high and low risk survival analysis in the nomogram model; **C**. ROC curves for prognostic predictive effect of the nomogram model; **D**–**F**. Calibration curves at 1, 3 and 5 years; **G**–**I**. DCA curves at 1, 3 and 5 years

After removing samples with missing values in clinicopathological parameters, a total of 374 cases were included in the construction of the nomogram model. A nomogram including age, T-stage, N-stage, and risk score was constructed (Fig. 4A). The scores based on the nomogram model were divided into a high-risk group and a low-risk group based on the median value of the scores. The OS in the high-risk group was significantly shorter (*P* < 0.05) (Fig. 4B). The AUC of ROC curve was 0.72 (sensitivity 0.68, specificity 0.7) (Fig. 4C). It indicates that the nomogram model had accurate predictive effect on OS in HNSC patients. Predictive analyses were performed for 1-, 3-, and 5 year periods for OSCC, demonstrating the nomogram possesses strong validation performance and predictive accuracy (Fig. 4D–I).

### GO, KEGG and GSVA analysis

A total of 643 genes were differentially expressed between the high and low risk score groups (Supplementary Fig. 3A). The GO enrichment analysis showed that the top five enriched BPs included signal release, regulation of membrane potential, hormone metabolic process, external encapsulating structure organization (Supplementary Fig. 3B); the top five enriched CCs included apical part of cell, collagen-containing extracellular matrix, synaptic membrane, apical plasma membrane, and endoplasmic reticulum lumen (Supplementary Fig. 3C); the top five enriched MFs included passive transmembrane transporter activity, channel activity, signaling receptor activator activity, receptor ligand activity, and ion channel activity (Supplementary Fig. 3D, Supplementary Table 5). The KEGG results showed that the main enriched pathways were Cytokine-cytokine receptor interaction, cAMP signaling pathway, Cell adhesion molecules, Cushing syndrome, and IL-17 signaling pathway (Supplementary Fig. 3E, Supplementary Table 6).

Using “h.all.v7.5.1.symbols.gmt” as the reference gene set, the results suggested that the enrichment levels of Epithelial Mesenchymal Transition, Angiogenesis, Apical Junction, Hypoxia, Coagulation and Glycolysis pathways were significantly higher in the high-risk group than in the low-risk group. In contrast, the enrichment levels of E2F Targets, DNA Repair, G2M Checkpoint and Oxidative Phosphorylation were significantly lower in the high-risk group than in the low-risk group (*P* < 0.05) (Supplementary Fig. 4A).

Using “c2.cp.kegg.v7.5.1.symbols.gmt” as the reference gene set, the results suggested that the enrichment levels of ECM Receptor Interaction, Focal Adhesion, Gap Junction, Wnt Signaling Pathway, and MAPK Signaling Pathway were significantly more enriched in the high-risk group than in the low-risk group. The enrichment levels of Butanoate Metabolism, Fatty Acid Metabolism, Primary Immunodeficiency, Selenoamino Acid Metabolism, and Nucleotide Excision Repair pathways were significantly lower in the high-risk group than in the low-risk group (*P* < 0.05) (Supplementary Fig. 4B).

### Risk score was associated with HNSC metabolism

**Fig.5:**
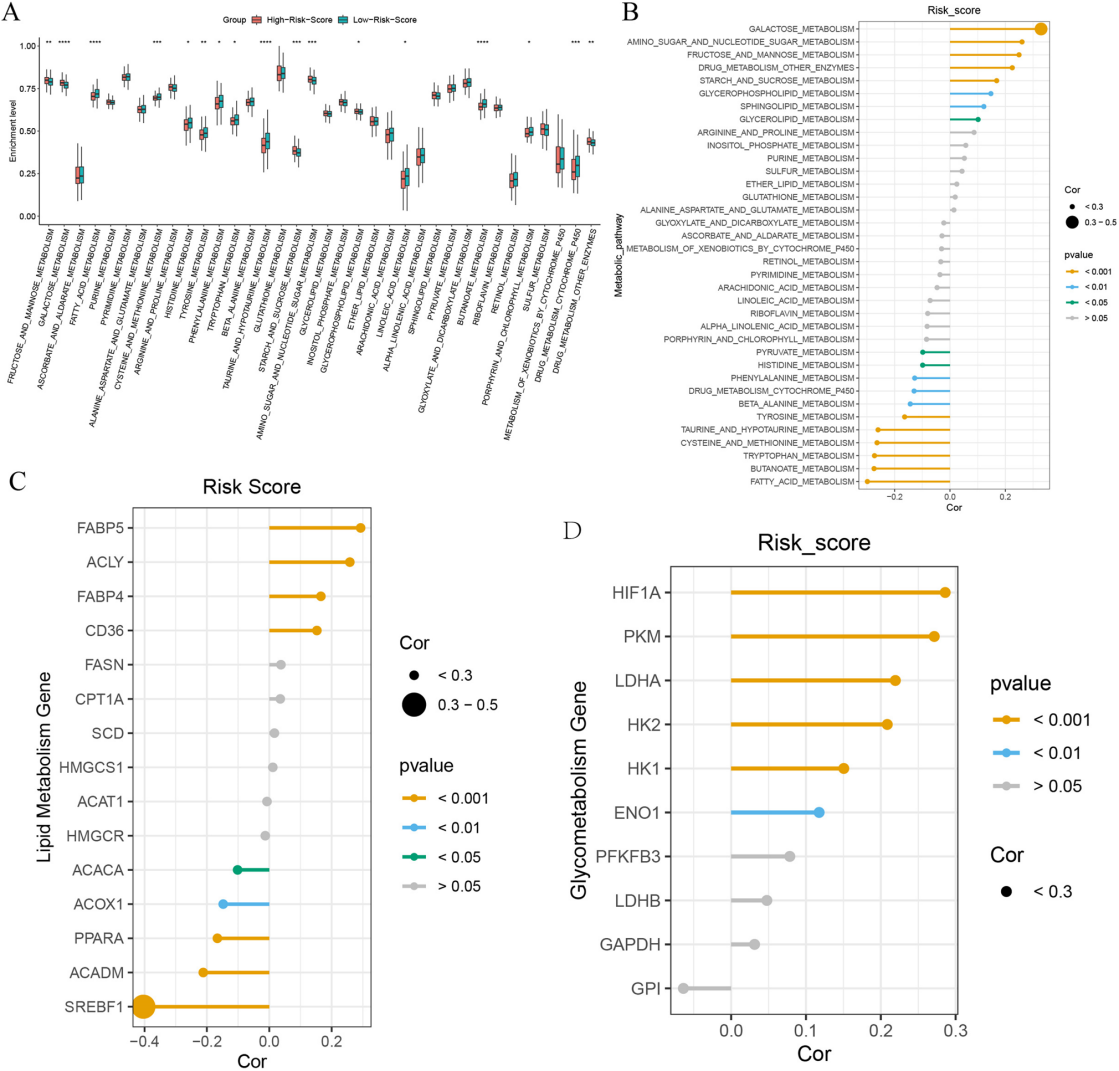
Metabolism-related pathway enrichment analysis and metabolism key gene expression analysis. **A**. Differences in metabolism-related pathway enrichment between high and low risk groups; **B**. Correlation between risk score and metabolism-related pathway enrichment level; **C**. Correlation between risk score and lipid metabolism-related gene expression level; **D**. Correlation between risk score and glucose metabolism-related gene expression level

To further understand the effect of risk score on the enrichment of metabolic pathways, 36 metabolism-related pathways ssGSEA were analyzed. The results suggested that glucose metabolism-related pathways were enriched at higher levels in the high-risk group, while lipid metabolism pathways were enriched at higher levels in the low-risk group (*P* < 0.05) (Fig. 5A). Risk score was significantly positively correlated with Galactose Metabolism and Amino Sugar And Nucleotide Sugar Metabolism pathway enrichment levels, and negatively correlated with Fatty Acid Metabolism pathway enrichment levels (*P* < 0.05) (Fig. 5B).

The risk score was significantly positively correlated with the expression levels of FABP5, ACLY, FABP4 and negatively correlated with the expression levels of SREBF1, ACADM and PPARA in lipid metabolism (*P* < 0.05) (Fig. 5C). The risk score was significantly and positively correlated with the expression levels of HIF1A, PKM, LDHA, HK1, HK2, and ENO1 in glucose metabolism (*P* < 0.05) (Fig. 5D).

### Risk score was associated with HNSC immunity

**Fig.6:**
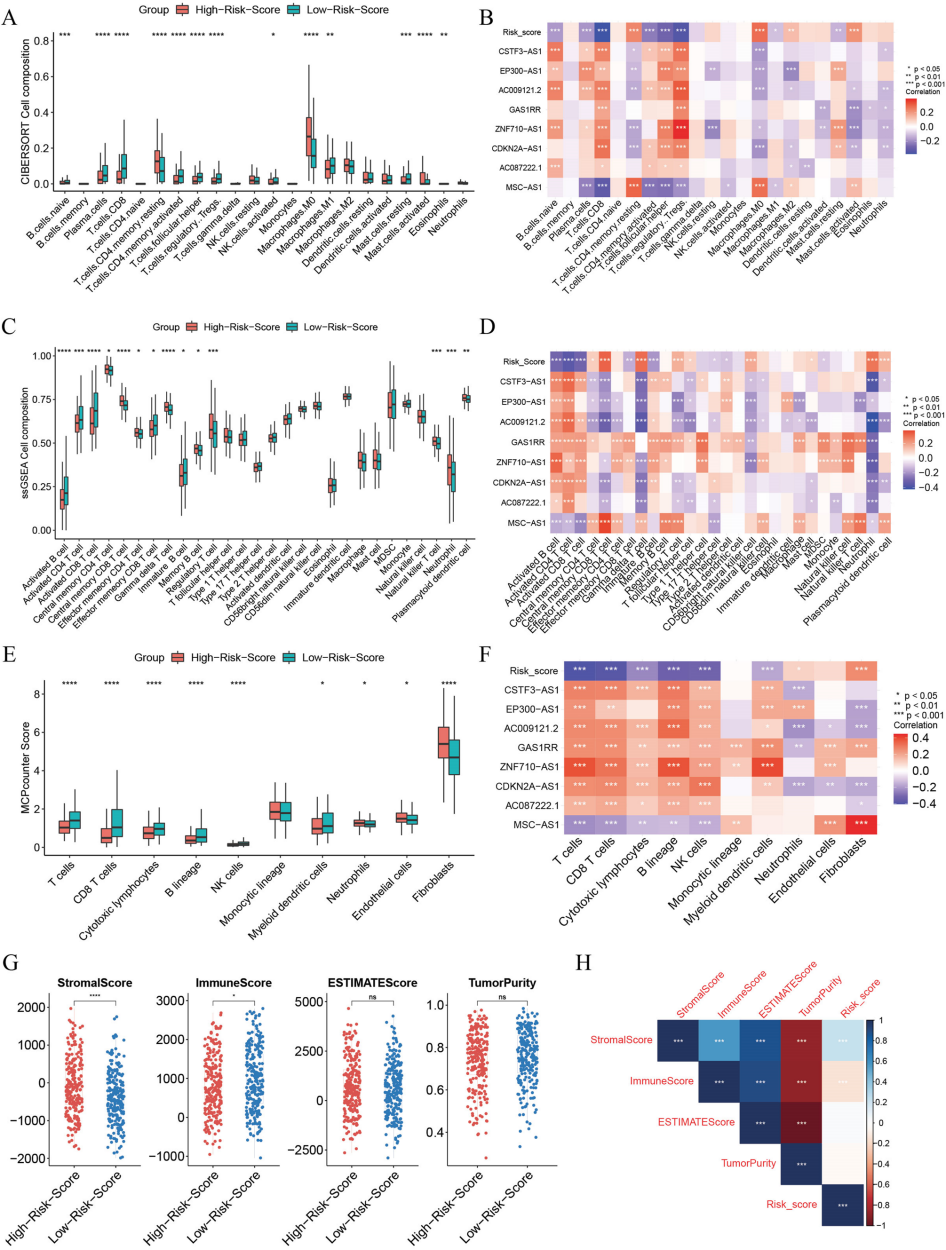
Immune cell infiltration and immune microenvironment analysis. **A**. Box plot of immune cell infiltration analysis by CIBERSORT method; **B**. Correlation of signature lncRNA and risk score with the level of immune cell infiltration by CIBERSORT; **C**. Box plot of immune cell infiltration analysis by ssGSEA method; **D**. Correlation of signature lncRNA and risk score with the level of infiltration of each immune cell in ssGSEA; **E**. Box plot of immune cell infiltration analysis by MCPcounter method; **F**. Correlation of signature lncRNA and risk score with the level of infiltration of each immune cell by MCPcounter; **G**. ESTIMATE immune microenvironment analysis; **H**. Correlation analysis of risk score with ESTIMATE immune microenvironment indicators

CIBERSORT, ssGSEA, and MCPcounter results showed that the infiltration levels of T cell, B cell and Macrophages were significantly higher in the high-risk group than in the low-risk group (*P* < 0.05). At the same time, lncRNAs and risk score were also correlated with immune cell infiltration to varying degrees (*P* < 0.05) (Fig. 6A–F).

ESTIMATE analysis showed that StromalScore was significantly higher in the high-risk group than in the low-risk group, while ImmuneScore was significantly lower than in the low-risk group (*P* < 0.05). Risk scores were significantly positively correlated with StromalScore and significantly negatively correlated with ImmuneScore (*P* < 0.05) (Fig. 6G, H). These results demonstrated that the risk score signature correlated with the HNSC immune microenvironment.

### The risk score was correlated with HNSC immunotherapy

**Fig.7:**
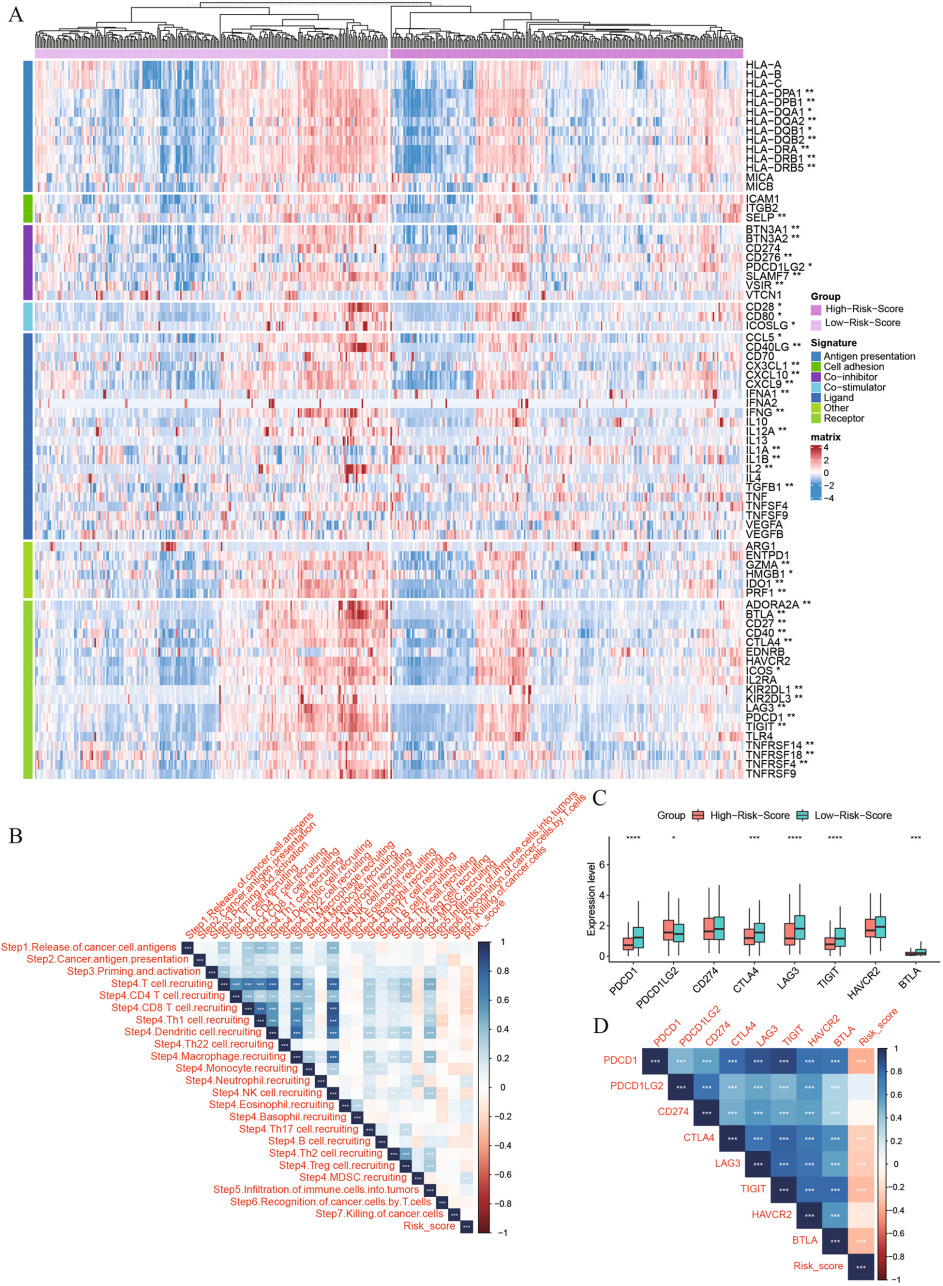
Immunotherapy analysis. **A**. Expression of immune-related genes in high- and low-risk groups; **B**. Correlation between risk score and each immunophenotype; **C**. Difference in expression of immune checkpoints between high- and low-risk groups; **D**. Correlation of risk scores and immune checkpoint expression levels

The expression levels of BTN3A1, BTN3A2, CD276, PDCD1LG2, SLAMF7, VISR in immunostimulatory genes and CD28, CD80, ICOSLG in immunosuppressive genes were significantly higher in the low-risk patients than in the high-risk group (*P* < 0.05) (Fig. 7A).

Correlation analysis of risk score with each immune event suggested that risk score was significantly negatively correlated with initiation and activation (Step 3) (*P* < 0.05). In immune cell recruitment to tumor (step 4), risk score was associated with T cell recruiting, CD4 T cell recruiting, CD8 T cell recruiting, Th1 cell recruiting, Th1 cell recruiting, Monocyte recruiting NK cell recruiting, Th17 cell recruiting, B cell recruiting and Treg cell recruiting were significantly negatively correlated, and Neutrophil recruiting, Eosinophil recruiting, Basophil recruiting and MDSC recruiting were significantly positively correlated (*P* < 0.05) (Fig. 7B). In addition, the expression levels of the immune checkpoint inhibitors PDCD1 (PD1), CTLA4, LAG3, TIGIT and BTLA were significantly lower in the high-risk group than in the low-risk group (Fig. 7C, D) (*P* < 0.05). These results suggested that risk scores can provide a reference when developing immunotherapy regimens.

### TMB analysis

In the high-risk group, the top five genes with the highest mutation frequency were TP53 (77%), TTN (35%), CDKN2A (23%), FAT1 (23%), and NOTCH1 (19%) (Supplementary Fig. 5A). On the other hand, in the low-risk group, the top five genes with the highest mutation frequency were TP53 (56%), TTN (37%), SYNE1 (21%), MUC16 (20%), and FAT1 (19%) (Supplementary Fig. 5B). The mutation rate of TP53 was significantly higher in the high-risk group compared to the low-risk group, and the mutation rates of SYNE1 and MUC16 were significantly lower in the high-risk group compared to the low-risk group (*P* < 0.05) (Supplementary Fig. 5C). KM analysis suggested that OS was significantly shorter in the high-risk high-TMB group than in the low-risk low-TMB group (*P* < 0.05) (Supplementary Fig. 5D,E).

### Drug sensitivity analysis

**Fig.8:**
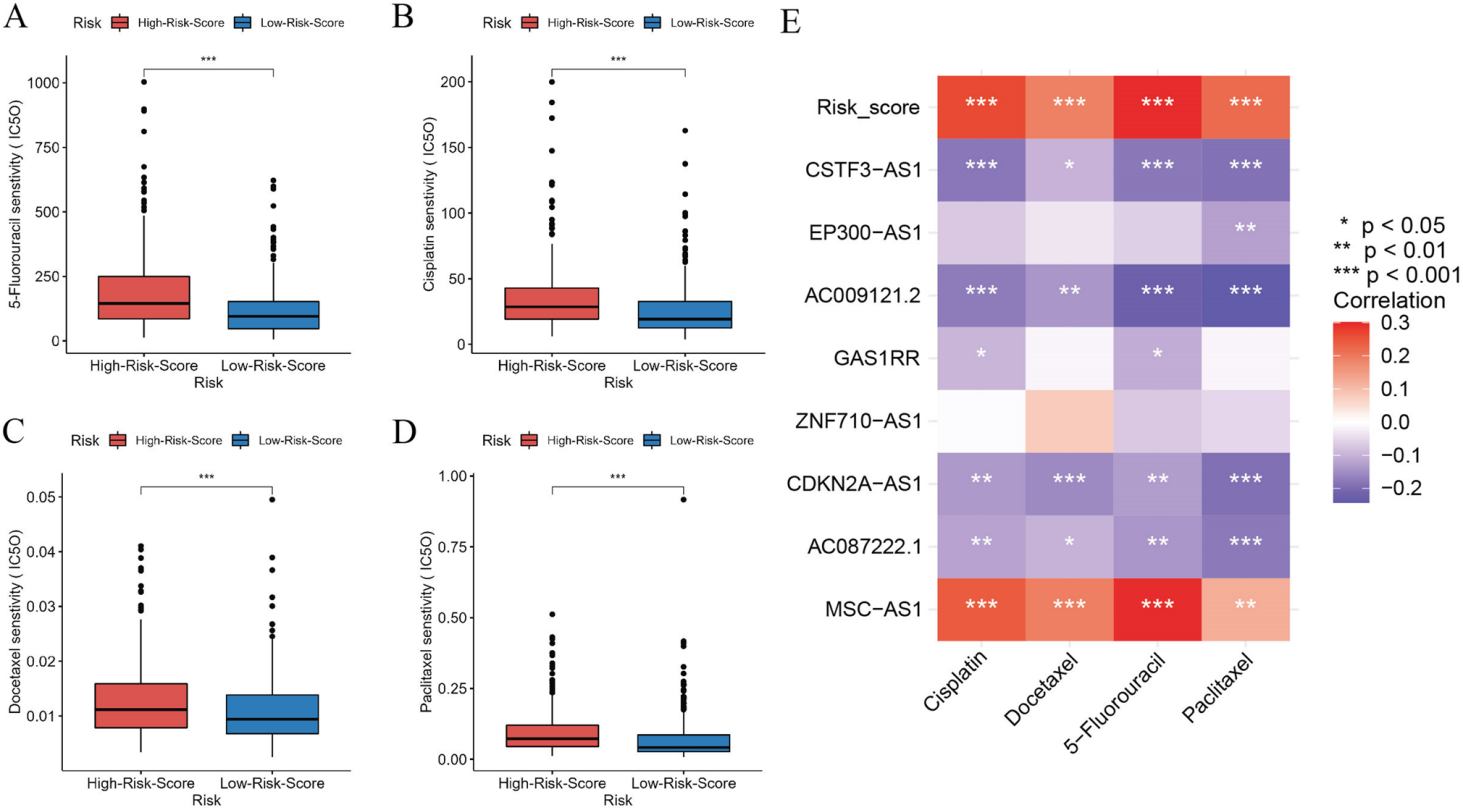
Drug sensitivity analysis. **A**–**D**. Differences in IC50 between high and low risk groups, A. 5-Fu, B. cisplatin, **C**. docetaxel, **D**. paclitaxel; **E**. Correlation of risk scores and signature lncRNA expression levels with IC50 of the four drugs

The IC50 of 5-Fu, cisplatin, docetaxel and paclitaxel was significantly higher (*P* < 0.05) in the high-risk group compared to the low-risk group (Fig. 8A–D). This suggests that the risk score can be used as a predictor of drug sensitivity in HNSC. The expression of MSC-AS1 was significantly positively correlated with the IC50 of all four drugs (*P* < 0.05). The expression of CSTF3-AS1, AC009121.2, CDKN2A-AS1 and AC087222.1 were significantly negatively correlated with the IC50 of the four drugs (*P* < 0.05) (Fig. 8E).

### Signature lncRNA clustering analysis

**Fig.9:**
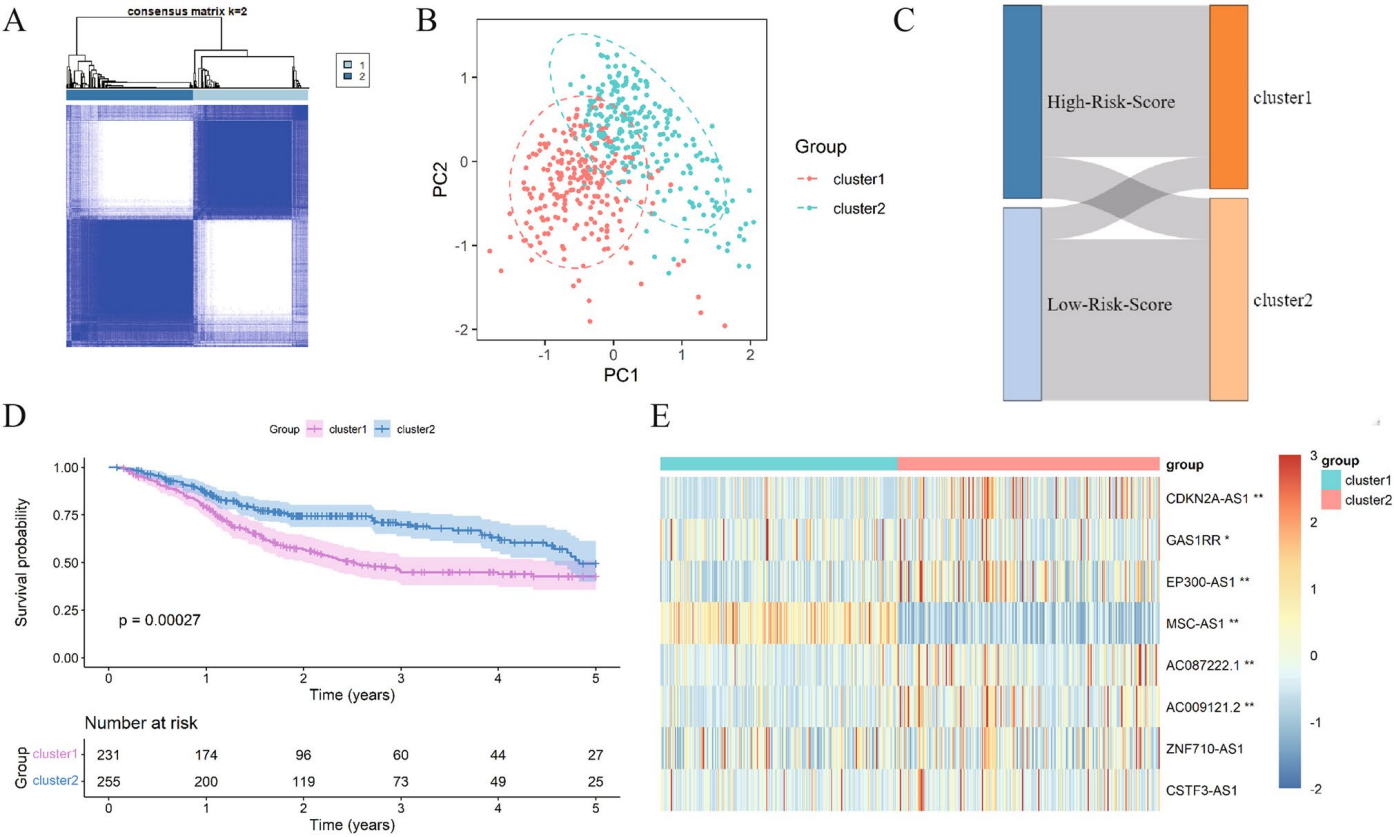
Cluster analysis. **A**. ConsensusCluster cluster plot; **B** PCA plot of cluster analysis; **C**. Sankey plot of sample consistency between high and low risk groups and cluster analysis; **D**. KM curves of survival analysis in different cluster groups; **E**. Heatmap of lncRNA expression in different clusters in the risk signature

Cluster analysis was performed to analyze the clustering effect of lncRNAs in signature. Based on the expression of the eight lncRNAs in the signature, the samples were divided into two clusters (Fig. 9A). Principal Component Analysis (PCA) further confirmed the clear distinction between these two clusters (Fig. 9B). The sankey diagram showed that cluster1 was significantly associated with the high-risk group, while cluster 2 was associated with the low-risk group (Fig. 9C). KM analysis showed that OS was significantly prolonged in cluster2 compared to cluster1 (*P* < 0.05) (Fig. 9D). The expression of CDKN2A-AS1, GAS1RR, EP300-AS1, MSC-AS1, AC087222.1 and AC009121.2 in signature lncRNAs was significantly different between cluster1 and cluster2 (*P* < 0.05) (Fig. 9E).

### RT-qPCR

**Fig.10:**
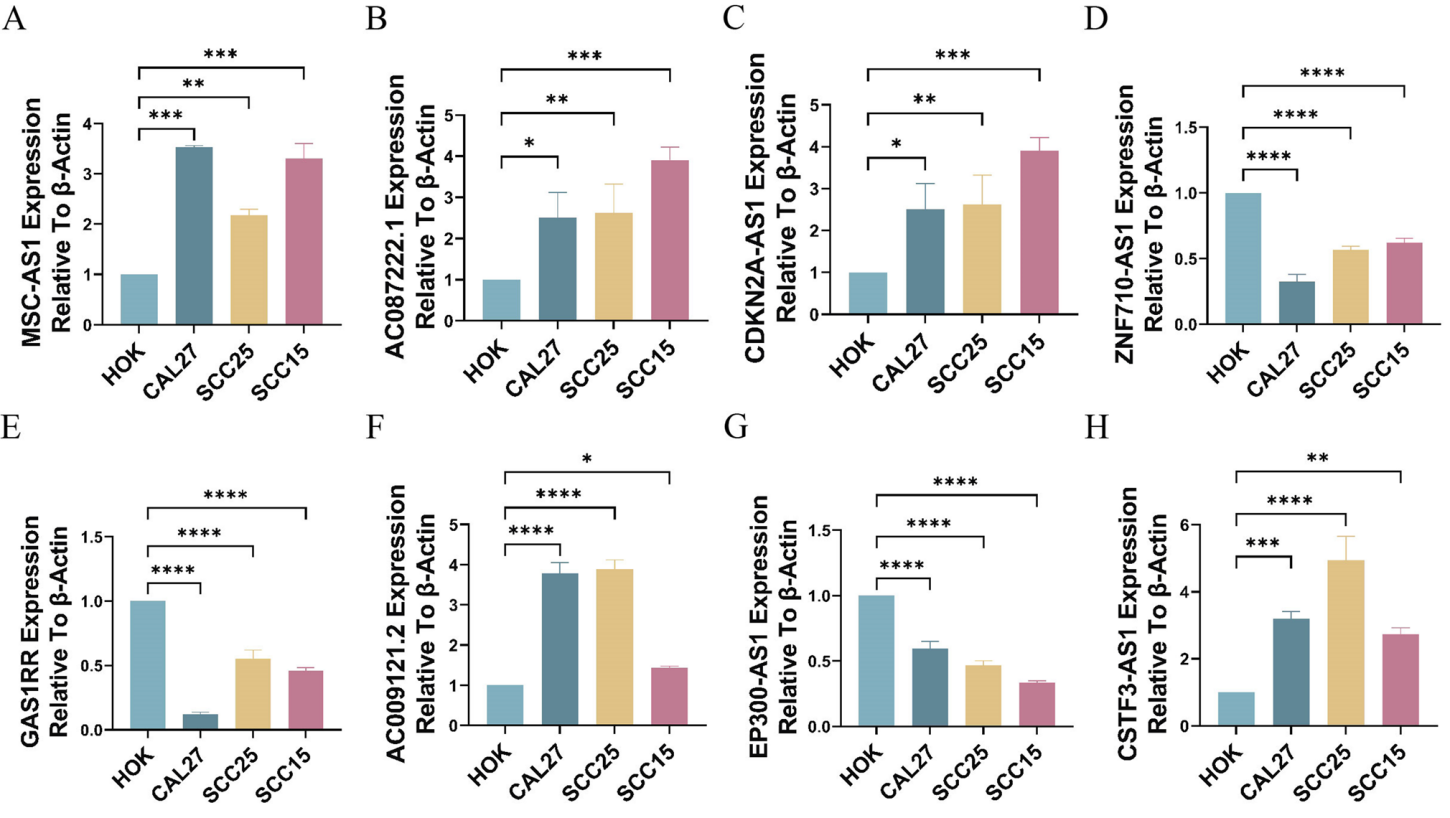
RT-qPCR to validate the expression of 8 signature lncRNAs. **A**–**G**. RNA expression of MSC-AS1 (**A**), AC087222.1 (**B**), CDKN2A-AS1 (**C**), ZNF710-AS1 (**D**), GAS1RR (**E**), AC009121.2 (**F**), EP300-AS1 (**G**), and CSTF3-AS1 (**H**). (*P < 0.05,**P < 0.01,***P < 0.001)

The results of RT-qPCR were consistent with the data in TCGA. MSC-AS1, AC087222.1, CDKN2A-AS1, AC009121.2 and CSTF3-AS1 had increased expression in HNSC cells, whereas ZNF710-AS1, GAS1RR, and EP300-AS1 had decreased expression in OSCC tumor cells (Fig. 10A–H).

## Discussion

The disruption of the circadian rhythm may promote the development of cancer. Some studies have suggested that night work and some circadian genes polymorphisms are associated with cancer [24]. In HNSC, the expression of circadian clock genes is also altered, especially the core circadian clock genes, and different genetic alterations are associated with different clinical parameters [25].

The available studies have demonstrated that lncRNAs are involved in cellular pathways associated with cancer and have good predictive power in terms of prognosis and diagnosis [26, 27]. Emerging studies are attempting to develop novel and effective risk signatures for lncRNAs in malignancies and to discover molecular features of malignancies and potential therapeutic targets [28–30]. Similar studies using lncRNA to construct prognosis-related signatures have been widely applied in HNSC [31, 32]. In this study, we developed a risk score signature using eight circadian rhythm-associated lncRNAs. The survival time of those with high-risk scores was shorter compared to low-risk scores. The results of multivariate COX regression analysis suggested that risk score was an independent risk factor for HNSC prognosis. These results illustrated that the risk score could be used as a marker to predict prognosis. In addition, the clustering analysis of HNSC tumor samples based on the expression of 8 lncRNAs in this study revealed that they could be better clustered into two clusters, showed good agreement with the grouping results between high and low risk score groups. Thus, the reliability of the signature consisting of these 8 lncRNAs was further demonstrated.

Some lncRNAs in the signature have been reported in various cancers. In the present study, MSC-AS1 was a risk factor for HNSC prognosis, and patients with high expression of MSC-AS1 had shorter survival time. Similarly, previous studies have demonstrated that MSC-AS1, as a risk factor, can promote the progression of colorectal cancer and also melanoma [33, 34]. The remaining seven lncRNAs in the signature were all protective factors for HNSC in this study, and the higher the expression level, the better the prognosis of HNSC. However, in previous studies CDKN2A-AS1 could promote the development of epithelial ovarian carcinoma [35], and overexpression of ZNF710-AS1 promoted the proliferation of clear cell renal carcinoma cells and inhibited apoptosis by regulating the expression of ZNF710 [36]. These findings are inconsistent with the signature results in the present study, which may be due to the different cancer species and require subsequent in-depth basic experimental studies.

We then analyzed the enrichment differences in common metabolic pathways in the high- and low-risk groups using the ssGSEA method. The results showed a higher level of enrichment in glucose metabolism-related pathways in the high-risk group, which may be related to the fact that glucose metabolism can promote the malignant progression of HNSC [37]. GSVA analysis of high- and low-risk groups showed a significant enrichment of epithelial-mesenchymal transition and angiogenesis, glycolysis and hypoxia-related pathways in the high-risk group, which were associated with tumor progression. Upregulation of Glycolysis in HNSC was associated with tumor progression [38]. Hypoxia was associated with poor prognosis in oral squamous cell carcinoma [39], and Zandberg et al. [40] found that Hypoxia was associated with resistance of HNSC to PD-1 blockade. Immunomodulation is crucial in the development, establishment, progression and treatment of HNSC. The results showed that the risk score was significantly positively correlated with the level of macrophage and neutrophil infiltration.

Among them, macrophages were the immune cells with the highest level of infiltration as suggested by CIBERSORT results, and M0 macrophages were considered to be associated with immunosuppression and worse prognosis in HNSC [41]. The expression levels of PDCD1 (PD1), CTLA4, LAG3,TIGIT and BTLA were significantly lower in the high-risk group than in the low-risk group, while patients with upregulated PD-1 usually responded better to consistent treatment with immune checkpoints [42]. LAG-3 was overexpressed on TIL in HNSC patients, and its expression correlated with high pathological grade, tumor size, and poorer prognosis [43]. The inhibition of TIGIT significantly delayed tumor growth and enhanced antitumor immune responses in a mouse model of HNSC [44]. These results suggest that the present study signature can provide a reference for immunotherapy of HNSC.

In the present study, the mutation rate was significantly higher in the high-risk group than in the low-risk group.TP53 mutations are an independent risk factor for HNSC prognosis [45]. TP53 mutation status can be used as a biomarker for postoperative adjuvant chemotherapy treatment decisions [46]. Furthermore, TP53 mutations can be used as predictors of HNSC response to immunotherapy [47, 48]. The 5-Fu, cisplatin, docetaxel and paclitaxel are commonly used in the treatment of HNSC. Koritala et al. [49] found that the effectiveness of cisplatin in tumor therapy in the presence of a biological clock is time-dependent. For recurrent and metastatic HNSC, a regimen of cisplatin and weekly docetaxel showed good efficacy and tolerable toxicity [50]. The IC50 of 5-Fu, cisplatin, docetaxel and paclitaxel was significantly lower in the high-risk group than in the low-risk group, and the risk score was significantly and positively correlated with the IC50 of these four drugs. It indicates that the signature can guide clinicians to select the appropriate chemotherapeutic agents for HNSC.

The present study has several limitations, firstly, this risk signature was built and validated based on TCGA data, and there is a lack of external validation data to provide more evidence for its application. Secondly, the mechanism of action of some lncRNAs in HNSC in the signature is still unclear. We will follow up with further in vitro and in vitro experimental studies.

## Conclusion

In conclusion, we analyzed significant correlations between lncRNA signature and OS. Cox analyses, stratified prognostic analyses, and ROC curves demonstrated that the signature was stable and accurate for prognostic analysis. It showed correlations with tumor metabolism, immune microenvironment and drug sensitivity, providing valuable insights into the treatment of HNSC.

### Supplementary Information

Supplementary File1. Fig. 1 KM curves of prognosis between high and low risk groups in different clinicopathologic parameter subgroups.

Supplementary File 2. Fig. 2 Predictive effect of signature lncRNAs on prognosis. A. KM curves for survival analysis of individual lncRNAs in the signature; B. Box plots of differential expression of individual lncRNAs in the signature.

Supplementary File 3. Fig. 3 DEG and its enrichment analysis between high and low risk groups. A. Volcano plot of genes differentially expressed between high and low risk groups; B. GO enrichment BP part of differentially expressed genes between high and low risk groups; C. GO enrichment CC part of differentially expressed genes between high and low risk groups; D. GO enrichment MF part of differentially expressed genes between high and low risk groups; E. KEGG enrichment analysis of differentially expressed genes between high and low risk groups.

Supplementary File 4. Fig. 4 GSVA analysis. A. GSVA analysis with h.all.v7.5.1.symbols.gmt as the reference gene set; B. GSVA analysis with c2.cp.kegg.v7.5.1.symbols.gmt as the reference gene set.

Supplementary File 5. Fig. 5 Tumor mutation burden analysis. A. Mutational gene waterfall plot in the high-risk group; B. Mutational gene waterfall plot in the low-risk group; C. Differences in mutation levels of TP53,TTN,CDKN2A,SYNE1,MUC16 between the high- and low-risk groups; D. Survival analysis KM curves for the risk-scored combined TMB; E Survival analysis KM curves between high-risk and low-risk high TMB and low TMB groups.

Supplementary File 6. Table S1 Circadian rhythm related genes.

Supplementary File 7. Table S2 Primer sequences for RT-qPCR.

Supplementary File 8. Table S3 Clinicopathological information of tumor samples.

Supplementary File 9. Table S4 the characteristics of HNSC patients in training cohort and validation cohort.

Supplementary File 10. Table S5 GO enrichment analysis.

Supplementary File 11. Table S6 KEGG enrichment analysis.

## Data Availability

The datasets analysed during the current research are all available in the UCSC-XENA database (https://xenabrowser.net/datapages/) and MsigDB (http://www.gsea-msigdb.org/gsea/index.jsp).
