# Supplementary figures and images for: A circadian rhythm-related lncRNA signature correlates with prognosis and tumor immune microenvironment in head and neck squamous cell carcinoma
Source: Discov Oncol. 2024 Jul 25;15:308. doi: 10.1007/s12672-024-01181-z (PMC11272767; doi:10.1007/s12672-024-01181-z)

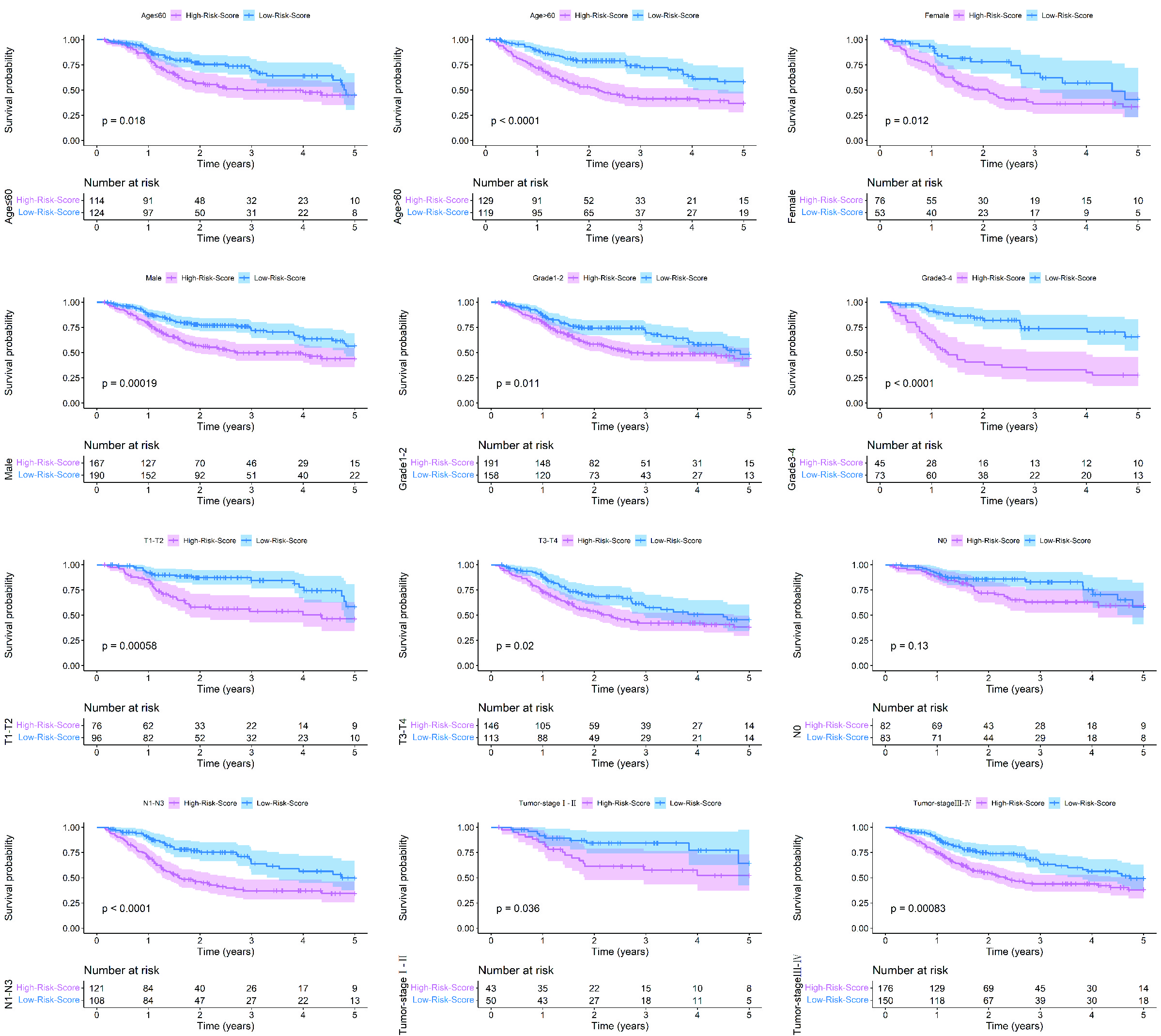

Supplement: Supplementary file 1 — Supplementary File1. Fig. 1 KM curves of prognosis between high and low risk groups in different clinicopathologic parameter subgroups. [file 12672_2024_1181_MOESM1_ESM.jpg]

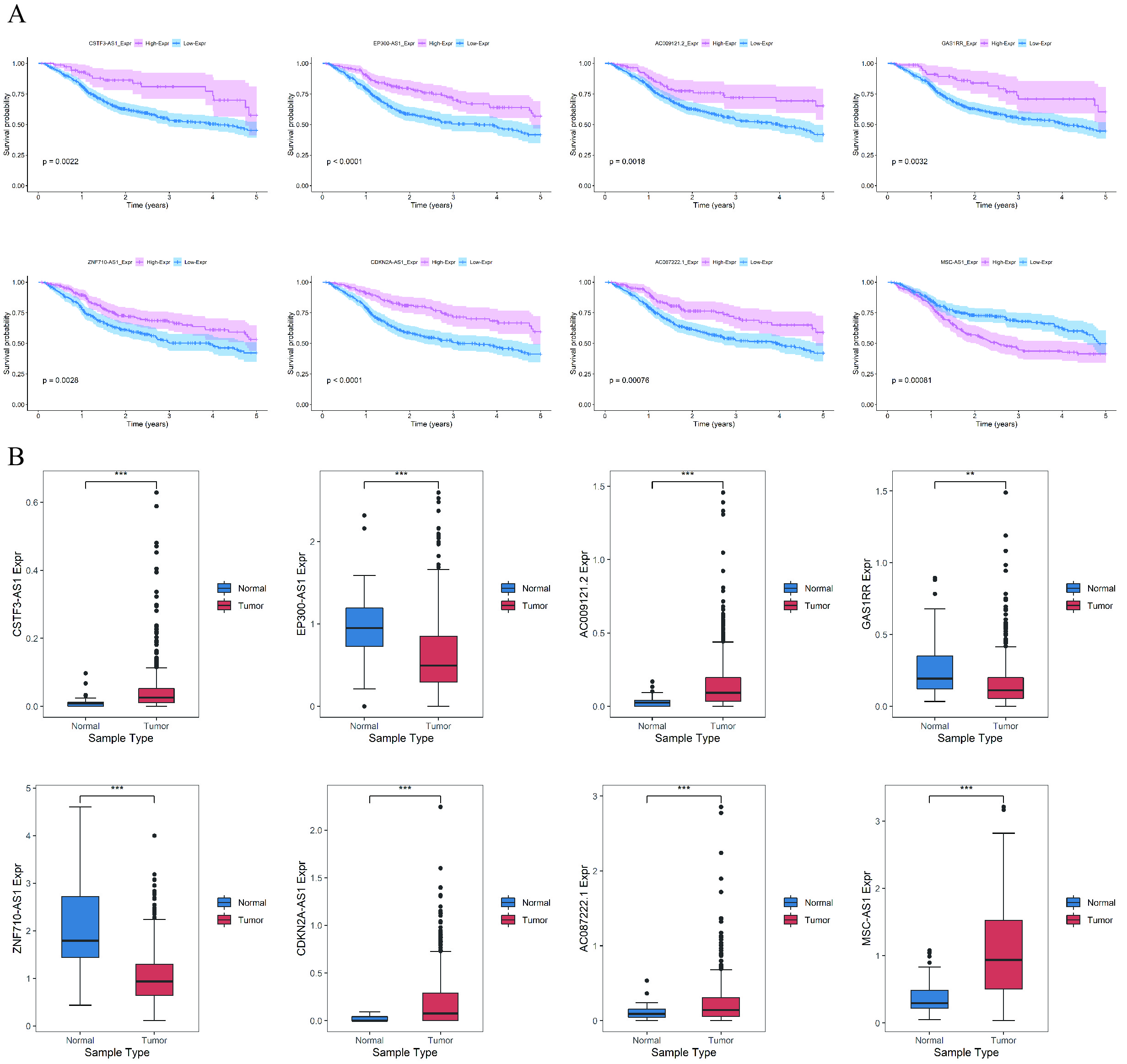

Supplement: Supplementary file 2 — Fig. 2 Predictive effect of signature lncRNAs on prognosis. A. KM curves for survival analysis of individual lncRNAs in the signature; B. Box plots of differential expression of individual lncRNAs in the signature. [file 12672_2024_1181_MOESM2_ESM.jpg]

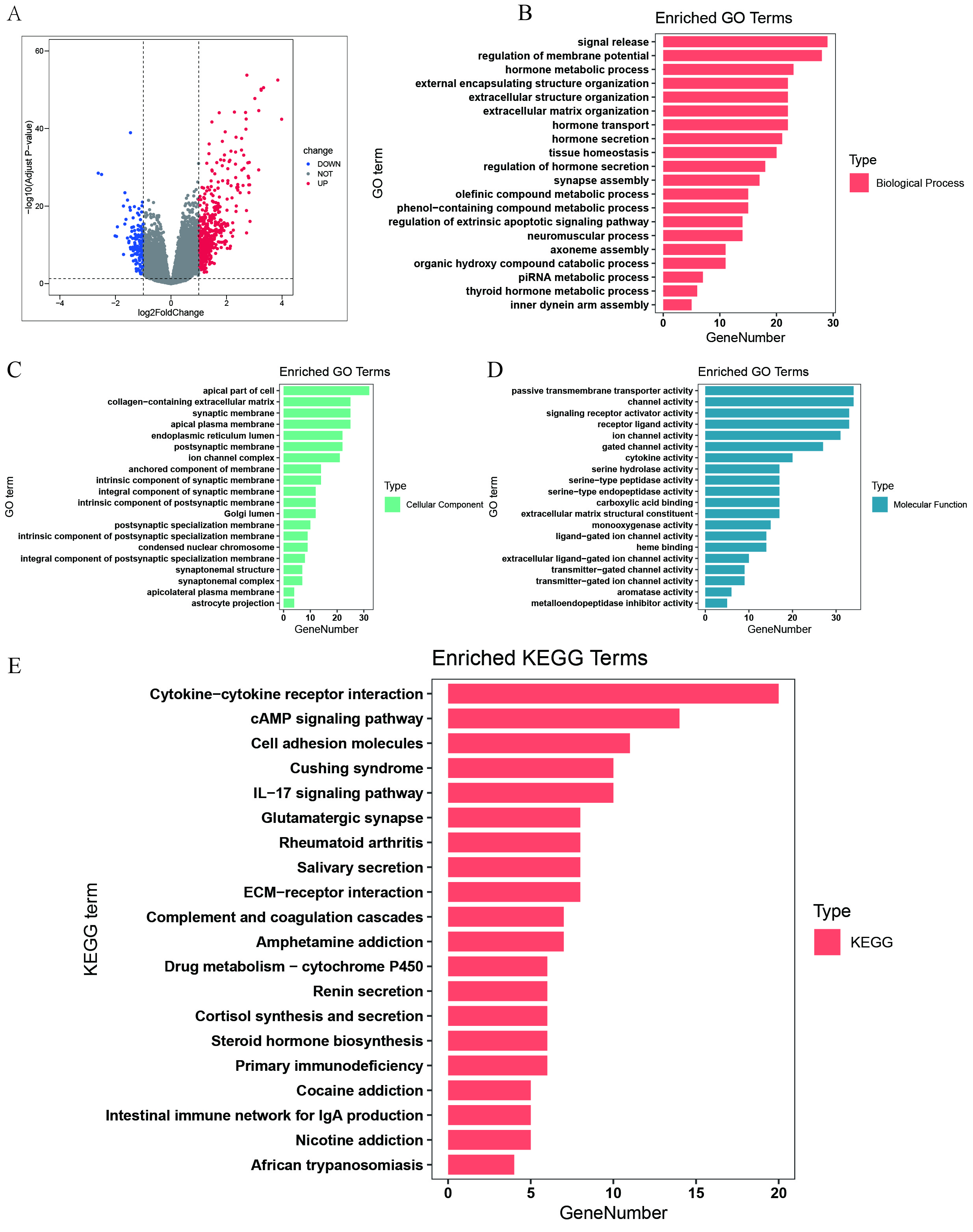

Supplement: Supplementary file 3 — Fig. 3 DEG and its enrichment analysis between high and low risk groups. A. Volcano plot of genes differentially expressed between high and low risk groups; B. GO enrichment BP part of differentially expressed genes between high and low risk groups; C. GO enrichment CC part of differentially expressed genes between high and low risk groups; D. GO enrichment MF part of differentially expressed genes between high and low risk groups; E. KEGG enrichment analysis of differentially expressed genes between high and low risk groups. [file 12672_2024_1181_MOESM3_ESM.jpg]

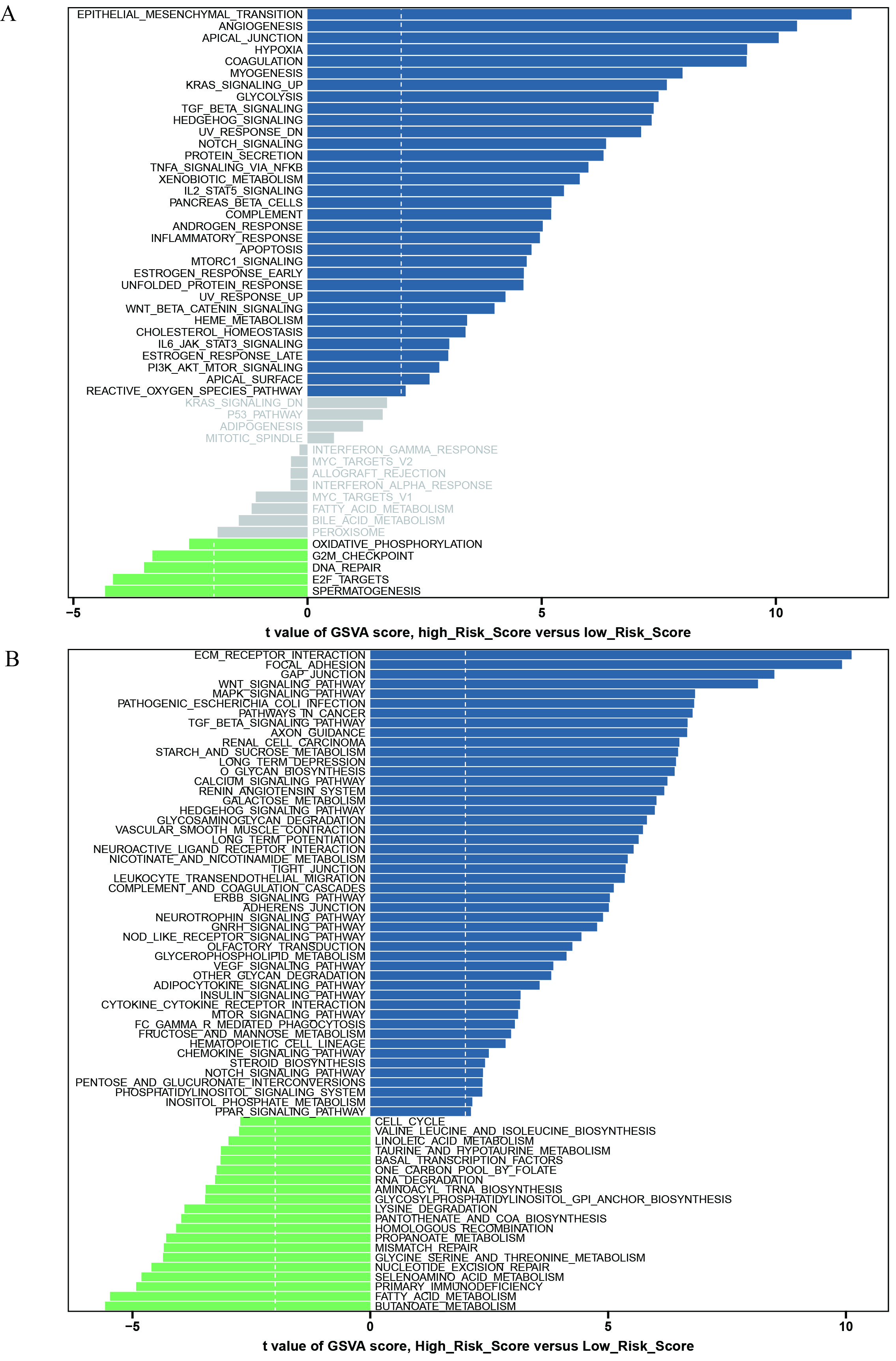

Supplement: Supplementary file 4 — Fig. 4 GSVA analysis. A. GSVA analysis with h.all.v7.5.1.symbols.gmt as the reference gene set; B. GSVA analysis with c2.cp.kegg.v7.5.1.symbols.gmt as the reference gene set. [file 12672_2024_1181_MOESM4_ESM.jpg]

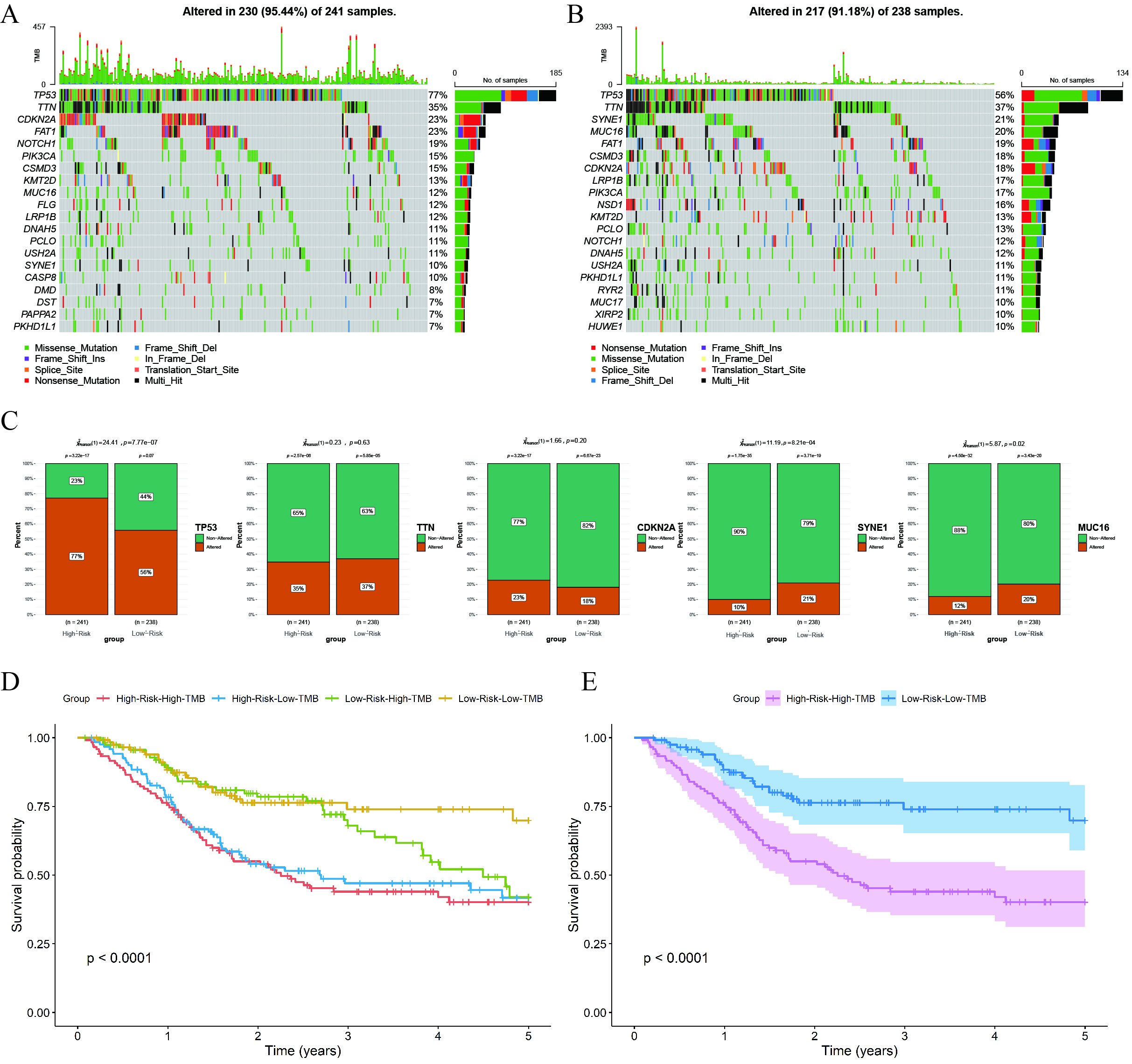

Supplement: Supplementary file 5 — Fig. 5 Tumor mutation burden analysis. A. Mutational gene waterfall plot in the high-risk group; B. Mutational gene waterfall plot in the low-risk group; C. Differences in mutation levels of TP53,TTN,CDKN2A,SYNE1,MUC16 between the high- and low-risk groups; D. Survival analysis KM curves for the risk-scored combined TMB; E Survival analysis KM curves between high-risk and low-risk high TMB and low TMB groups. [file 12672_2024_1181_MOESM5_ESM.jpg]
