# Supplementary material for: A circadian rhythm-related lncRNA signature correlates with prognosis and tumor immune microenvironment in head and neck squamous cell carcinoma
Source: Discov Oncol. 2024 Jul 25;15:308. doi: 10.1007/s12672-024-01181-z (PMC11272767; doi:10.1007/s12672-024-01181-z)
Supplement: Supplementary file 6 — Table S1 Circadian rhythm related genes. [file 12672_2024_1181_MOESM6_ESM.docx]

**Table S1**. Circadian rhythm related genes

| **Gene** | **Description** |
| --- | --- |
| CSNK1D | Casein Kinase 1 Delta |
| CSNK1E | Casein Kinase 1 Epsilon |
| PER1 | Period Circadian Regulator 1 |
| PER2 | Period Circadian Regulator 2 |
| PER3 | Period Circadian Regulator 3 |
| CRY1 | Cryptochrome Circadian Regulator 1 |
| CRY2 | Cryptochrome Circadian Regulator 2 |
| ARNTL | Aryl Hydrocarbon Receptor Nuclear Translocator Like |
| CLOCK | Clock Circadian Regulator |
| NPAS2 | Neuronal PAS Domain Protein 2 |
| NR1D1 | Nuclear Receptor Subfamily 1 Group D Member 1 |
| RORA | RAR Related Orphan Receptor A |
| RORB | RAR Related Orphan Receptor B |
| RORC | RAR Related Orphan Receptor C |
| BHLHE40 | Basic Helix-Loop-Helix Family Member E40 |
| BHLHE41 | Basic Helix-Loop-Helix Family Member E41 |
| RBX1 | Ring-Box 1 |
| CUL1 | Cullin 1 |
| SKP1 | S-Phase Kinase Associated Protein 1 |
| BTRC | Beta-Transducin Repeat Containing E3 Ubiquitin Protein Ligase |
| FBXW11 | F-Box And WD Repeat Domain Containing 11 |
| FBXL3 | F-Box And Leucine Rich Repeat Protein 3 |
| PRKAA1 | Protein Kinase AMP-Activated Catalytic Subunit Alpha 1 |
| PRKAA2 | Protein Kinase AMP-Activated Catalytic Subunit Alpha 2 |
| PRKAB1 | Protein Kinase AMP-Activated Non-Catalytic Subunit Beta 1 |
| PRKAB2 | Protein Kinase AMP-Activated Non-Catalytic Subunit Beta 2 |
| PRKAG1 | Protein Kinase AMP-Activated Non-Catalytic Subunit Gamma 1 |
| PRKAG3 | Protein Kinase AMP-Activated Non-Catalytic Subunit Gamma 3 |
| PRKAG2 | Protein Kinase AMP-Activated Non-Catalytic Subunit Gamma 2 |
| CREB1 | CAMP Responsive Element Binding Protein 1 |
| TIMELESS | Timeless Circadian Regulator |
