# Supplementary material for: A circadian rhythm-related lncRNA signature correlates with prognosis and tumor immune microenvironment in head and neck squamous cell carcinoma
Source: Discov Oncol. 2024 Jul 25;15:308. doi: 10.1007/s12672-024-01181-z (PMC11272767; doi:10.1007/s12672-024-01181-z)
Supplement: Supplementary file 7 — Table S2 Primer sequences for RT-qPCR. [file 12672_2024_1181_MOESM7_ESM.docx]

**Table S2**. Primer sequences for RT-qPCR

| Gene | Direction | Sequence (5ʹ-3ʹ) |
| --- | --- | --- |
| MSC-AS1 | forward | ACGTAGCCGTTCTCATAGCG |
|  | reverse | CCTTGGACGTGGCAGGTATT |
| AC087222.1 | forward | TGCTGCCTTAGCGAGGAAAA |
|  | reverse | GTGTCCCGTTTTGTAGCACG |
| CDKN2A-AS1 | forward | GAGGCCTGGTGAGCAAAATA |
|  | reverse | AAAGCCGTGTCTCAAGATCG |
| ZNF710-AS1 | forward | CTGTATGAGGGAGGTGGGGA |
|  | reverse | AGGAACCAGAGCAGAACGTG |
| GAS1RR | forward | AAGGAAAGAGATGCCTGGCC |
|  | reverse | GGCAACCAAGGTAAGCTCCT |
| AC009121.2 | forward | AACTCTCCGTGGGCTCAATG |
|  | reverse | CGGGGTTAACAGAGGGAACC |
| EP300-AS1 | forward | CAGAGCGGGAGGAGCG |
|  | reverse | CGGGGGGCAGAGGTTG |
| CSTF3-AS1 | forward | GCAAGCATTTCTCGCCTACG |
|  | reverse | AACCAGTCCTCCATTGCTGG |
| β-Actin | forward | CGTGACATTAAGGAGAAGCTG |
|  | reverse | CTAGAAGCATTTGCGGTGGAC |
