# Supplementary material for: A circadian rhythm-related lncRNA signature correlates with prognosis and tumor immune microenvironment in head and neck squamous cell carcinoma
Source: Discov Oncol. 2024 Jul 25;15:308. doi: 10.1007/s12672-024-01181-z (PMC11272767; doi:10.1007/s12672-024-01181-z)
Supplement: Supplementary file 8 — Table S3 Clinicopathological information of tumor samples. [file 12672_2024_1181_MOESM8_ESM.docx]

**Table S3**. Clinicopathological information of tumor samples

| **Characteristic** | **Freq** |
| --- | --- |
| Age | 61 ( 53 to 68.75 ) |
| Gender |  |
| female | 129 (27%) |
| male | 357 (73%) |
| Pathologic_T |  |
| T1 | 43 (9%) |
| T2 | 129 (28%) |
| T3 | 95 (21%) |
| T4 | 164 (35%) |
| TX | 32 (7%) |
| Pathologic_N |  |
| N0 | 165 (36%) |
| N1 | 65 (14%) |
| N2 | 157 (34%) |
| N3 | 7 (2%) |
| NX | 68 (15%) |
| Pathologic_M |  |
| M0 | 177 (75%) |
| MX | 60 (25%) |
| Grade |  |
| G1 | 60 (13%) |
| G2 | 289 (62%) |
| G3 | 116 (25%) |
| G4 | 2 (0%) |
| Tumor_stage |  |
| Stage Ⅰ | 24 (6%) |
| Stage Ⅱ | 69 (16%) |
| Stage Ⅲ | 78 (19%) |
| Stage Ⅳ | 248 (59%) |
