# Supplementary material for: A circadian rhythm-related lncRNA signature correlates with prognosis and tumor immune microenvironment in head and neck squamous cell carcinoma
Source: Discov Oncol. 2024 Jul 25;15:308. doi: 10.1007/s12672-024-01181-z (PMC11272767; doi:10.1007/s12672-024-01181-z)
Supplement: Supplementary file 9 — Table S4 the characteristics of HNSC patients in training cohort and validation cohort. [file 12672_2024_1181_MOESM9_ESM.docx]

**Table S4**. The characteristics of HNSC patients in training cohort and validation cohort.

| **Characteristics** | **Train** | **Test** | **χ^2^** | ***P* value** |
| --- | --- | --- | --- | --- |
| Age |  |  | 1.3828 | 0.2396 |
| ≤60 | 113 | 125 |  |  |
| > 60 | 132 | 116 |  |  |
| Gender |  |  | 0.091115 | 0.7628 |
| Female | 67 | 62 |  |  |
| Male | 178 | 179 |  |  |
| T stage |  |  | 6.3876 | 1.172 |
| T1 | 22 | 21 |  |  |
| T2 | 68 | 61 |  |  |
| T3 | 37 | 58 |  |  |
| T4 | 85 | 79 |  |  |
| TX | 19 | 13 |  |  |
| N stage |  |  | 2.0928 | 0.7187 |
| N0 | 80 | 85 |  |  |
| N1 | 28 | 37 |  |  |
| N2 | 80 | 77 |  |  |
| N3 | 4 | 3 |  |  |
| NX | 37 | 31 |  |  |
| M stage |  |  | 0.72533 | 0.3944 |
| M0 | 81 | 96 |  |  |
| MX | 23 | 37 |  |  |
| Grade |  |  | 1.1402 | 0.7674 |
| G1 | 30 | 30 |  |  |
| G2 | 140 | 149 |  |  |
| G3 | 63 | 53 |  |  |
| G4 | 1 | 1 |  |  |
| Tumor stage |  |  | 0.82377 | 0.8438 |
| Ⅰ | 13 | 11 |  |  |
| Ⅱ | 36 | 33 |  |  |
| Ⅲ | 36 | 42 |  |  |
| Ⅳ | 120 | 128 |  |  |
| Survival status |  |  | 0.23505 | 0.6278 |
| Alive | 137 | 141 |  |  |
| Death | 108 | 100 |  |  |
